# A Case of Early Osteoarthritis in a Patient With Ehlers-Danlos Syndrome

**DOI:** 10.7759/cureus.27069

**Published:** 2022-07-20

**Authors:** Sydney Tran, Radhika Thakkar, Monica Gillie, Jeffrey Anderson

**Affiliations:** 1 Orthopedic Surgery, St. George's University, St. George, GRD; 2 Medicine, St. George's University, St. George, GRD; 3 Family Medicine, Stanford Health Care, San Jose, USA; 4 Orthopedic Surgery, O'Connor Hospital, San Jose, USA

**Keywords:** pain management, salter osteotomy, connective tissue disorder, hypermobile disorders, osteoarthritis (oa), ehlers danlos syndrome

## Abstract

We present a case of early onset osteoarthritis in a patient with Ehlers-Danlos syndrome (EDS) and a history of developmental dysplasia of the hip. Ehlers-Danlos syndrome (EDS) is part of a wide spectrum of connective tissue disorders characterized by hyperextensible skin, hypermobile joints, and tissue fragility. Presentation varies from mild hyperextensibility of the skin and joints to debilitating physical disabilities and vascular complications because of genetic defects in type one and three collagen synthesis. Collagen is the most abundant protein in nearly all parts of the body and errors in the production of this protein have widespread effects. Therefore, we suggest a multidisciplinary approach to the management of patients with EDS, with an emphasis on patient education, to aid in the prevention and early detection of complications.

## Introduction

Ehlers-Danlos syndrome (EDS) is a part of a wide spectrum of connective tissue disorders characterized by hyperextensible skin, hypermobile joints, and tissue fragility. These classic symptoms are present in varying degrees in all subtypes of EDS and can lead to complications like chronic pain from joint hyperlaxity [1]. Early recognition and management with a multidisciplinary team are crucial to maximizing quality of life. Because there is no cure for EDS, patient education should also be prioritized for prompt recognition of possible complications. This case report presents a case of Ehlers-Danlos syndrome associated with developmental dysplasia of the hip with early onset osteoarthritis and discusses the management of other possible complications.

## Case presentation

A now 24-year-old female with a known history of classical EDS and developmental dysplasia of the hip status post-Salter Osteotomy first presented to the orthopedic office in 2015 with a chief complaint of gradually worsening back pain. The pain was described as dull and constant and rated a six out of ten with radiation from the neck down to the lower back. It was not associated with any specific injury and is minimally relieved with ibuprofen and physical therapy. 

On physical exam, the patient had bilateral paraspinal tenderness to palpation of the neck as well as the lumbar region at the level of L4-L5. The patient had a full range of motion of the neck and back and straight leg raise was negative bilaterally, and she was neurologically within normal limits. The right hip was noted to have a slight restricted internal rotation but there was no leg length discrepancy. The right knee was tender over the prepatellar area with no noticeable effusion leading to the clinical diagnosis of cervical and lumbar paraspinal strain and contusion and prepatellar bursitis of the right knee. Further imaging or laboratory work was deemed unnecessary at that time.

**Figure 1:**
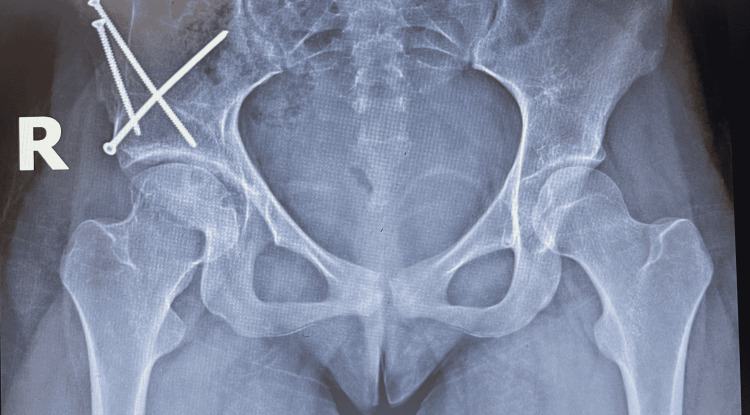
Prior Salter osteotomy of the right hip as evidenced by three screws in the pelvis. The right femoral head is elongated and flattened with a notable subchondral cyst. Articular cartilage is narrowed bilaterally with joint space loss greater on the left.

A follow-up appointment three months later revealed worsening hip pain localized to the right groin. The patient reported marked difficulty in bearing weight on the right lower limb and now had a limping gait that favored the left lower limb. Both active and passive motion of the right hip was severely restricted, particularly with an internal rotation where a range of motion was limited to an estimated 15 degrees. X-ray imaging revealed a grossly normal thoracolumbar spine; however, there was a notable deformity of the right hip seen in Figure 1. History of a previous Salter osteotomy performed by a different orthopedic surgeon was evidenced by three screws in her pelvis with an asymmetric right femoral head performed approximately ten years earlier with a completed follow-up course. The femoral head was elongated and flattened with a subchondral cyst. The articular space was narrowed bilaterally with nonuniform joint space loss where the left demonstrated greater loss than the right. Overall, the radiographic findings were consistent with severe osteoarthritis of the hip. The plan now involved non-steroidal anti-inflammatory drugs (NSAIDs) for hip pain. Weight-bearing exercises such as running and jumping were discouraged and the patient was instructed to return to the office as needed.

Further visits all followed a similar framework of addressing her chronic pain with medications, physical therapy, and home exercises. More recently, the patient developed bilateral foot drop, weakness with ankle dorsiflexion, and the inability to heel walk. An MRI and neurology consult were inconclusive with suspicion of a left S1 perineural root sleeve cyst. Despite compliance with recommended interventions such as NSAIDs and physical therapy, the pain has persisted and continues to limit her mobility and quality of life.

## Discussion

Ehlers-Danlos syndrome is a complex diagnosis requiring a collaborative approach in management that should include patient education. A core team should include a geneticist, cardiologist, ophthalmologist, psychiatrist/psychologist, orthopedic surgeon, physical/occupational therapist, and a primary care physician. In addition to regular visits with the above team members, patients should be aware of specific symptoms, such as in this case of osteoarthritis, so that interventions can take place earlier on in the disease process to improve patient quality of life.

Although the true prevalence of EDS is unknown, a UK study in 2019 saw 553 patients diagnosed with EDS in a sample size of 1,298 [2]. The pathophysiology of this group of disorders is also unclear and varies with each of the subtypes: Classical EDS is characterized by a COL5A1 or COL5A2 mutation, while the vascular subtype is caused by a COL3A1 mutation [3,4]. Because the vascular subtype mutation affects type three collagen, it is the most severe form of EDS with spontaneous rupture of organs leading to a shorter life span [4]. While there are multiple other subtypes, we focus on the classic, hypermobile and vascular subtypes here as they are the most common [3].

Patients with classical EDS have hyperextensible, fragile skin with joint hypermobility. Hyperextensibility refers to skin that is able to stretch beyond the normal range and recoils back once released [4]. Other classic features include thin, atrophic scars over areas prone to injury (forehead, elbows, knees, etc.) epicanthal folds, easy bruisability, and flat feet with hallux deformities [5]. The hypermobile subtype is similar in presentation to the classical form but without the tissue fragility, meaning scars and easy bruisability may not be present. Finally, with the vascular subtype, the defect in type three collagen leads to weak vessel walls that are prone to rupture with thin, translucent skin. Characteristic facial features include a thin nose, tight skin, hollow cheeks, and prominent eyes [4].

As EDS symptoms can manifest anywhere collagen is present and collagen is the most abundant protein in most areas of the body, management must address multiple systems, including patient education, emotional or psychological support, and minimizing pain and possible complications [6]. For acute and chronic pain control: opioids, massages, splints/braces, heat therapy, and avoidance of dangerous activities are perceived to be the most effective by patients [7]. The case discussed above previously made use of NSAIDs, splints/braces, and avoidance of contact sports to manage the patient's chronic pain. Complication management includes a baseline echocardiogram which is recommended in both children and adults; especially if the patient has the vascular subtype of EDS. Follow-up examinations every two to five years are suggested for the classical or hypermobile subtypes [8].

Joint protection is a major concern as orthopedic interventions are associated with a list of operative and postoperative complications including difficulty making incisions, wound dehiscence, and extensive bleeding from difficulty cauterizing or ligating friable vessels [9]. Hypermobility of the hip joint leads to instability and dysfunction that is associated with early onset osteoarthritis prompting surgical intervention [2,10]. Patients with EDS have a higher rate of periprosthetic dislocation and shorter implant survival after a total hip arthroplasty [11,12].

The case above demonstrates the need for early orthopedic involvement in EDS patients. An earlier Salter osteotomy would have likely prevented or postponed the patient’s early onset osteoarthritis as the patient’s femoral head was malformed because of prolonged, uncorrected developmental dysplasia of the hip. Due to the severity of the resulting osteoarthritis, we anticipate the patient will require a total hip arthroplasty within the next three years. Early patient education that emphasizes the protection of joints to preserve function is exceptionally important in patients with EDS, with an understanding that no pain is gained as the chronic joint instability and altered collagen in cartilage may cause early degenerative joint disease [13]. Non-weight-bearing exercises such as swimming or biking are encouraged, while running, jumping, and gymnastics should be avoided. For symptomatic pain relief, NSAIDs, acetaminophen, or opioids may be used, with adjuvant topical medications and joint injections.

Surgical intervention should be used cautiously on EDS patients as it is associated with a higher risk of complications, such as poor wound healing and dislocations. We suggest early management and education for joint protection in patients with EDS to avoid the need for surgical intervention. If that treatment option cannot be avoided, alternative approaches like dual-mobility components for a total hip arthroplasty should be considered to reduce the rate of dislocations [11]. Although knowing the subtype of EDS can help guide the management of symptoms, protocols for all types of EDS are similar and should be followed to provide the patient with the most pain relief possible, despite their incurable diagnosis. In summary, we presented a case of early onset osteoarthritis in a patient with EDS to encourage early collaborative and preventative management of any subtype of EDS to optimize therapeutic options and prevent late recognition of OA.

Alarming symptoms that EDS patients should be aware of include sudden onset chest pain or orthostatic hypotension as these can be signs of an acute vascular event such as aortic dissection. Although vascular accidents are feared, acute chest pain could also be attributed to rib-sternal subluxations and costochondritis from cartilaginous laxity [14,15]. Of note, the FDA states that fluoroquinolone antibiotics should be avoided as they are associated with an increased risk of aortic rupture [16].

## Conclusions

As Ehlers-Danlos syndrome affects many systems within the body, a multidisciplinary management approach should be prioritized with early orthopedic involvement. Special attention to the hip joint should be given as surgical intervention is associated with a higher rate of postoperative complications such as poor wound healing and dislocations. Although the subtype of EDS may be used to direct treatment, management is similar and should follow the outline.
